# Epidemiological Trends of Carbapenemase-Producing *Pseudomonas aeruginosa* in a Tertiary Care Hospital in Athens, Greece, During 2020–2023 †

**DOI:** 10.3390/antibiotics14090898

**Published:** 2025-09-05

**Authors:** Vasiliki Koumaki, Eleni Voudanta, Aikaterini Michelaki, Maria Orfanidou, Eleni Vagiakou, Georgia Vrioni, Athanasios Tsakris

**Affiliations:** 1 Department of Microbiology, Medical School, National and Kapodistrian University of Athens, 115 27 Athens, Greece; elvoudanta@med.uoa.gr (E.V.); gvrioni@med.uoa.gr (G.V.); atsakris@med.uoa.gr (A.T.); 2 Department of Microbiology, General Hospital G. Gennimatas, 115 27 Athens, Greece; k.mixelaki@paidon-agiasofia.gr (A.M.); m.orfanidou@gna-gennimatas.gr (M.O.); mikrobiology@gna-gennimatas.gr (E.V.)

**Keywords:** carbapenemase-producing *Pseudomonas aeruginosa*, epidemiological trends, Greece, antimicrobial resistance, metallo-β-lactamases

## Abstract

**Background:** Infections caused by carbapenemase-producing *Pseudomonas aeruginosa* (CPPA) isolates have become a worldwide clinical challenge for clinicians due to the limited treatment options. This study provides epidemiological data on CPPA clinical isolates recovered from one of the largest tertiary care hospitals in Athens, Greece, serving a diverse patient population during and after the COVID-19 pandemic. **Materials and Methods**: The study included all consecutive single-patient CPPA clinical isolates identified from January 2020 to December 2023 in the clinical laboratory. Identification and antimicrobial susceptibility testing were performed using the VITEK-2 automated system. A lateral flow immunoassay and the FilmArray system for blood cultures only were used for the detection of the five most prevalent carbapenemases. Their epidemiological and antimicrobial susceptibility trends were retrospectively analyzed. **Results:** During the study period, a total of 628 single-patient CPPA were identified among 902 carbapenem-resistant *P. aeruginosa* clinical isolates. An increasing number of CPPA was revealed during the survey, especially in the post-COVID period (rising from *n* = 102 in 2020, *n* = 105 in 2021, and *n* = 123 in 2022 to *n* = 298 in 2023; *p* < 0.05). Regarding the type of carbapenemase, VIM metallo-beta-lactamase was the only carbapenemase identified during the first two years of the study (2020 to 2021). In 2022, VIM- and NDM-producing isolates split almost evenly at proportions of 57% and 43%, respectively. In 2023, NDM-producing isolates seem to have surpassed VIM producers with rates of 54.4% and 45.6%, respectively. As far as antimicrobial resistance profiles, high rates of resistance were observed for most of the antipseudomonal drugs, exceeding 90% across all study years, with little significant variation. However, aztreonam exhibited moderate activity and colistin exhibited excellent activity, remaining the most viable drugs in this setting. **Conclusions:** Following the COVID-19 pandemic, an increase in CPPA pathogens was identified, while an epidemiological shift was also observed, with the carbapenemase NDM dominating over VIM since 2023. Continuous surveillance is required to track resistance patterns and guide empirical therapy. In this context, new antimicrobials and antimicrobial combinations are also urgently needed.

## 1. Introduction

*Pseudomonas aeruginosa* constitutes one of the major threats in hospital-acquired infections globally, associated with high morbidity and mortality rates [1,2,3,4]. The pathogen is commonly associated with healthcare infections, particularly in hospitalized patients. These include lower respiratory tract infections (especially severe for patients with chronic lung diseases), bloodstream infections, urinary tract infections, and surgical site infections [2,4,5]. *P. aeruginosa* belongs to the ESKAPE group of pathogens, which also includes *Enterococcus faecium, Stapylococcus aureus, Klebsiella pneumoniae, Acinetobacter baumanni,* and *Enterobacter* spp. Although carbapenem-resistant *Pseudomonas aeruginosa* (CRPA) has been reclassified from a “critical” to a “high”-priority pathogen according to the World Health Organization (WHO) Bacterial Priority Pathogen List, it remains a serious public health concern [6].

Moreover, the global rise in infections due to CRPA isolates presents a significant public health threat and a major challenge for clinicians due to the limited treatment options available, as these organisms possess resistance against a wide spectrum of antimicrobials. Notably, infections caused by carbapenemase-producing *P. aeruginosa* (CPPA) are associated with increased 30-day mortality rates compared to non-carbapenemase-producing isolates, highlighting the significant clinical burden imposed by these multidrug-resistant pathogens [7].

A variety of different resistance mechanisms can occur simultaneously in *P. aeruginosa*, including the loss of the OprD porin, the increased production of efflux pumps, over-expression of the intrinsic AmpC-type cephalosporinase, and production of antibiotic-inactivating enzymes such as β-lactamases [8]. Carbapenem resistance in *P. aeruginosa* was often considered to be attributed to OprD deficiency and, less frequently, to carbapenemase production [9]. However, this situation has been changing during the last decade, and several reports have documented a widespread dissemination of carbapenemases among CRPA isolates.

Notably, carbapenemases are not intrinsically expressed by *P. aeruginosa*, but rather acquired by horizontal gene transfer and can be easily disseminated [5]. These enzymes are frequently co-expressed with other β-lactamases (various extended-spectrum β-lactamases [ESBLs] and/or AmpC β-lactamases), complicating the treatment further [1]. MBLs are frequently located in integrons that may carry additional resistance genes, facilitating the spread of resistance [4,10].

Therefore, carbapenem resistance in *P. aeruginosa* often coexists with resistance to the major classes of antibiotics, including β-lactams, aminoglycosides, quinolones, and polymyxins [4,11,12], leaving clinicians with limited therapeutic options, such as novel β-lactam/β-lactamase inhibitors, polymyxin- or fosfomycin-based combinations [13], and monobactams like aztreonam [4].

CRPA can harbor a variety of carbapenemases such as NDM (New Delhi metallo-β-lactamase), IMP (imipenemase), VIM (Verona integron-encoded metallo-β-lactamase), OXA-48-like carbapenemases, and KPC (*Klebsiella pneumoniae* carbapenemase). All documented types of transferable carbapenemases, except for SIM-1 (Seoul imipenemase), have been identified in *P. aeruginosa* isolates globally. Among these, metallo-β-lactamases (MBLs) are regarded as the most clinically significant [10,12].

The prevalence and distribution of these carbapenemases vary globally, with certain types being more predominant in specific regions. According to epidemiological data, VIM-type MBLs are commonly found in Europe and Asia, while NDM-type enzymes are more prevalent in South Asia and parts of Europe [3]. In China and South Central America, KPC and VIM were the predominant carbapenemase genes, while in Australia and Singapore, the predominant genes were NDM and IMP. In the Middle East, VIM and GES represent the most commonly encountered carbapenemase genes [7]. According to recent local data, MBLs constitute the major resistance mechanism in Greece [10]. Since the first outbreak of *P. aeruginosa* isolates harboring blaVIM isolated in AHEPA University Hospital of Thessaloniki, during the period of 1996–1998 [14], VIM has been the most prevalent mechanism among CRPA in Greece. However, since the emergence of the first NDM isolation in 2023, a rapid spread of NDM-producing isolates seems to have overcome VIM in some regions [15]. To our knowledge, sporadic epidemiological data have been published over the past few decades concerning CRPA infections, primarily focusing on Central and Northern Greece [14,15,16,17,18,19].

This study aims to record the current epidemiology of carbapenemases in clinical strains of CRPA and assess the antimicrobial resistance patterns in strains isolated from a tertiary care hospital located in the capital city, Athens, Greece, within the 2020–2023 timeframe.

## 2. Results

A total of 628 CPPA were identified among 902 CRPA single-patient isolates recovered in the clinical lab during the study period. More specifically, 102 CP strains were collected in 2020, 105 strains in 2021, 123 strains in 2022, and 298 strains in 2023. Isolates originated from various clinical specimens, including blood, urine, bronchial secretions, wound swabs, and other sample types (ocular specimens, drainage fluids, aspirates, etc.) Among them, urine and bronchial secretions were the most frequent samples, accounting for 39% and 20% of the total isolates, over the study period, respectively. Blood cultures and central venous catheter (CVC) samples accounted for smaller portions of 7% and 5%, respectively, with CVC showing a slight increase over time (Table 1).

**Figure 1:**
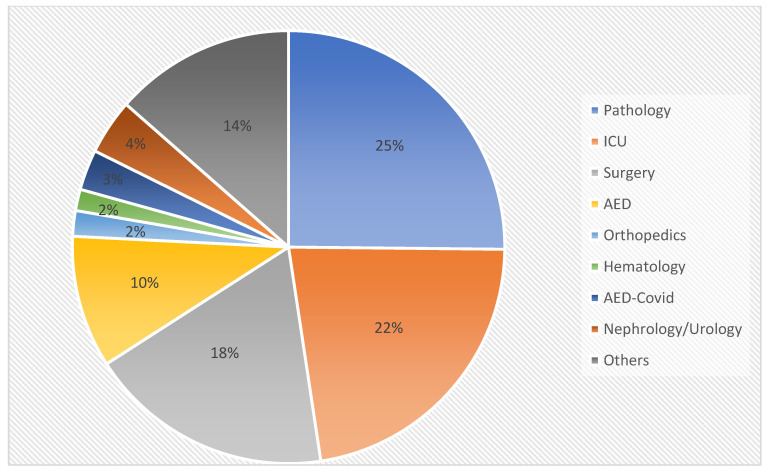
Distribution of carbapenemase-producing *P. aeruginosa* isolates by hospital department.

The clinical samples examined were gathered from numerous medical departments of the hospital, including Orthopedics, Pathology, Surgery, Hematology, ICU, Accident and Emergency Department (AED), AED-COVID, Nephrology/Urology, and others (gastroenterology, neurology, rheumatology, ophthalmology, thalassemia department, intermediate care unit, etc.). Across all four years of the study period (2020–2023), the Pathology department contributed the highest number of CPPA isolates, accounting for 25%, followed by the ICU department with 22%, and the Surgery department with 18% (Table 2, Figure 1).

A statistically significant increasing trend in CPPA isolates was observed during the study period, rising from *n* = 102 in 2020, *n* = 105 in 2021, *n* = 123 in 2022, to *n* = 298 in 2023 (*p* < 0.05). Regarding the carbapenemase type, a significant transition was observed during the studied period. In 2020 and 2021, all carbapenemase-producing isolates were VIM-positive. However, in 2022, an almost split emerged of VIM- and NDM-producing isolates, accounting for 57% and 43%, respectively. By 2023, this trend continued with a slight predominance of NDM-positive isolates, representing 54.4%, compared to 45.6% VIM-positive isolates. KPC-type, IMP, or OXA-48-like carbapenemase-producing strains were not detected during the whole study period (Table 3).

Table 4 presents the resistance rates of *P. aeruginosa isolates* (resistant isolates/total isolates tested) to the tested antibiotics from 2020 to 2023. Resistance rates remained consistently high, often approaching 100% with little significant variation. Of note, resistance rates to piperacillin/tazobactam, ceftazidime, cefepime, aztreonam and gentamicin increased significantly from 2020 to 2023.

Regarding aminoglycosides, the resistance rate to gentamicin was 80%, 96.1%, 93.9%, and 97.4%; tobramycin was 96.1%, 98.1%,99.2%, and 97.6%; and amikacin was 84.4%, 91.6%, 98.3%, and 92.3% for each consecutive year. Resistance to ciprofloxacin and levofloxacin significantly increased at percentages of 91.8%, 99%, 97.5%, 94.7%, and 92.6% and 99%, 97.6%, and 94.3%, respectively, for the given timeframe. Cephalosporin resistance rose evidently: ceftazidime—77.5%, 100%, 100%, and 99.7%; cefepime—85.3%, 98.1%, 99.2%, and 99%, indicating a statistically significant increase during the period studied. Ceftazidime/avibactam resistance was 100% for the years 2021, 2022, and 2023, while for 2020, no data was available. Piperacillin/tazobactam resistance increased from 80.4% in 2020 to 100% in subsequent years.

Resistance rate to colistin remained low, with rates of 2.9%, 1.9%, 1.6%, and 2.7% for the years 2020, 2021, 2022, and 2023, respectively. Aztreonam exhibited a gradual increase from 16.8%, 24.8%, 39.8% and 41.9%over the four years.

Table 5 details the antimicrobial resistance profiles of *P. aeruginosa* harboring VIM or NDM carbapenemases over the period 2020–2023. A continuous increase in the resistance rates of *Pseudomonas* spp. for both VIM and NDM was observed. For both mechanisms of resistance, the total percentage was over 85% for almost all antimicrobial agents. In VIM-positive isolates, aztreonam resistance was higher in NDM-positive isolates (55.1%) compared to VIM-positive isolates (23.8%). Colistin resistance rate remained low in both groups, at 1.9% for VIM-positive isolates and 3.3.% for the NDM-positive ones. Fluoroquinolone resistance remained above 88% during the whole period, with ciprofloxacin and levofloxacin showing 100% resistance in NDM strains in 2023. As for aminoglycosides, gentamicin and amikacin resistance was also high, notably 90.5% and 89.2% in VIM strains and 100% and 98.6% in NDM strains, respectively, in the whole study period. Resistance to piperacillin/tazobactam, ceftazidime and cefepime was constantly high among both VIM- and NDM-producing *P. aeruginosa* isolates, reaching 100% in NDM across all years.

## 3. Discussion

CPPA constitutes an emerging public health threat globally, primarily due to the lack of effective therapeutic options. *P. aeruginosa* exhibits intrinsic resistance to a broad range of antimicrobial agents [5] through intrinsic and acquired mechanisms [8]. In Greece, the prevalence of CRPA has reached critically high levels, posing a serious challenge for the healthcare system as treatment options remain extremely limited.

This study aimed to investigate the epidemiological trends and antimicrobial resistance profiles of CRPA clinical strains isolated from a tertiary care hospital in Athens over a four-year period (2020 to 2023). A total of 628 carbapenemase-producing *Pseudomonas* spp. clinical strains were examined during this period at the Microbiology Department of the General Hospital of Athens “Georgios Gennimatas”. The total number of isolates increased sharply over the study period, with a notable increase from 102 in 2020 to 298 in 2023, especially in the post-COVID period (*p* < 0.05).

Globally, the distribution of carbapenemase types in *P. aeruginosa* varies by region. Our findings demonstrate the widespread presence of MBLs among CRPA isolates collected during the study period. Notably, all CRPA isolates recovered in this survey harbored MBL enzymes, underscoring the dominance of this resistance mechanism in our setting. These results align with previous reports highlighting the high prevalence of MBL among CPPA in Greece [14,15,16,17,18].

The most remarkable change observed during the study period was the shift in carbapenemase distribution. NDM-positive strains appeared in 2022, comprising 43% of the total isolates, and by 2023, NDM-positive strains had become more prevalent, accounting for 54.4% of the total isolates. The first isolation of NDM-producing *P. aeruginosa* in Greece took place at the University Hospital of Thessaly in 2023 [10]. Since then, the incidence of NDM infections has steadily increased, revealing a transition from VIM- to NDM-producing *Pseudomonas* spp., associated with the spread of multidrug-resistant NDM-1-producing *P. aeruginosa* allocated to international high-risk clones, such as ST773 and ST308 in the Attica region [15,18]. This epidemiological trend is consistent with our current findings.

Urinary tract infections are among the most prevalent healthcare-associated infections, with *P. aeruginosa* being a significant pathogen, especially in hospitalized patients. Our study is in accordance with this finding, as urine was the most frequently encountered specimen of CPPA isolates. Infections caused by CRPA are associated with prolonged hospital stays, increased mortality, and higher healthcare costs. A retrospective analysis of hospitalized adults with urinary infections reported that 4.4% had infections due to carbapenem-resistant pathogens, with *P. aeruginosa* responsible for 49.4% of these cases. [20].

The increasing number of CRPA worsens the already difficult situation regarding the treatment of these infections. Our results indicate that *Pseudomonas* spp. exhibit a high level of resistance to almost all the antimicrobial agents tested in the Microbiology Department of “Georgios Gennimatas” General Hospital, as part of the clinical laboratory routine, with few exceptions. Colistin and aztreonam were identified as “the last resort” antibiotics, exhibiting lower resistance rate, i.e., 2.9%, 1.9%, 1.6%, 2.7% and 16.8%, 24.8%, 39.8%, 41.9%, respectively, for each year studied. Similarly, when stratified by the carbapenemase type, colistin and aztreonam remained potential treatment options for MBL-positive isolates. However, due to the unavailability of new drugs in our hospital and the country, coupled with bureaucracy in attaining them from abroad, infections due to MBL-positive *P. aeruginosa* isolates were mainly treated with combination therapy of ceftazidime/avibactam and aztreonam during the study period.

Ambler class B β-lactamases indeed confer a pan-β-lactam resistance phenotype except for aztreonam, high carbapenemase activity, and resistance to β-lactamase inhibitors [3,5]. VIM-type carbapenemases inactivate almost all β-lactams, leaving out aztreonam, while NDM-type enzymes hydrolyze β-lactams, including aztreonam, likely due to additional resistance mechanisms. Cefiderocol may also be an option for MBLs, although it is not yet available in Greece. Nevertheless, resistance to cefiderocol in *P. aeruginosa* isolates has been observed so far among NDM-producing isolates.

However, access to these drugs (aztreonam/avibactam and cefiderocol) remains limited globally. Aztreonam/avibactam was recently approved by the European Medicines Agency (EMA) in 2024 [21] and by the United States Food and Drug Administration (FDA) in 2025 [22]. However, it is not yet commercially available in most countries [23]. On the other hand, cefiderocol was approved by the FDA in 2019 and by the EMA in 2020 [24]. Despite its clinical potential, access to cefiderocol also remains limited, due to high acquisition costs and pricing limitations, especially in Asian and African regions [25,26].

The novel β-lactamase inhibitors do not inactivate MBLs, rendering therefore new β-lactam/β-lactamase inhibitor combinations, such as ceftolozane/tazobactam, ceftazidime/avibactam, imipenem/relebactam, and meropenem/vaborbactam inactive against both VIM and NDM carbapenemases. When active, amikacin and fosfomycin may be used to treat urinary tract infections [27,28]. Although amikacin, compared to other aminoglycosides, is usually a poor substrate for these enzymes and is recognized for exhibiting a stronger antibiotic activity against *P. aeruginosa* [4], our findings showed a high resistance level to amikacin also. On the other hand, fosfomycin was not tested in our study, since no EUCAST breakpoint for *P. aeruginosa* is available. Although colistin remains a last resort antimicrobial agent for the treatment of VIM and NDM carbapenemase-producing *Pseudomononas* spp., in alignment with our findings, its use should be guided by confirmatory genotypic and MIC results [15,29] and is limited by nephrotoxicity and neurotoxicity [30,31]. Careful dosing and monitoring are recommended to minimize risks and preserve colistin’s effectiveness [32].

The COVID-19 pandemic has worsened the situation by increasing antibiotic use and reducing the monitoring of multi-resistant pathogens, leading to high CRPA prevalence in hospital departments. A significant rise in multidrug-resistant organisms isolated from bloodstream infections during the pandemic has been reported, highlighting the crucial need for stringent surveillance and infection control measures [33].

The limitations of our study include the fact that only the antibiotics available in our hospital were evaluated. Novel antimicrobial agents, i.e., cefiderocol and newer β-lactam/β-lactamase inhibitor combinations, except for ceftazidime avibactam, were not included in the survey, as they had not been introduced in Greece during the study period. These agents may offer an additional therapeutic option for treating infections caused by CPPA.

Finally, despite the fact that our data are limited to a single hospital in Athens, although one of the biggest in Athens, an increasing spread of NDM-producing *P. aeruginosa* is evident. Continuous surveillance, effective antimicrobial stewardship, and research are essential to address the evolving epidemiology and improve prevention and treatment strategies.

## 4. Materials and Methods

### 4.1. Hospital Setting

This epidemiological study was conducted at one of the largest public tertiary care hospitals in Greece-located in Athens, the capital city and a central hub of healthcare. The hospital serves a diverse patient population and maintains a structured microbiological database, making it a suitable site for surveillance of antimicrobial resistance trends. It has a bed capacity of 726 beds and a bed occupancy rate of 90.3% over the study period. The retrospective analysis took place at the Department of Microbiology of the General Hospital “Geogios Gennimatas” over a four-year period from 2020 to 2023. All consecutive single-patient CRPA clinical isolates obtained during this timeframe at the Department of Microbiology were included. CRPA were defined as *P. aeruginosa* that exhibited resistance to at least one carbapenem (imipenem or meropenem) based on the EUCAST criteria for minimum inhibitory concentration (MIC) interpretive breakpoints. The isolates originated from various specimen types, including blood, urine, bronchial secretions, and central venous catheter (CVC) samples, wound samples, and others. The “other” category included a variety of less common but clinically significant samples, such as ocular specimens, drainage fluids, aspirates, etc. Isolates were obtained from both inpatients and outpatients across all hospital departments, such as Orthopedics, Pathology, Surgery, Hematology, Intensive Care Unit (ICU), Accident and Emergency Department (AED), AED-COVID, Nephrology/Urology, and others. The “Others” category encompasses more specialized and less common departments, such as the gastroenterology, neurology, rheumatology, ophthalmology, and thalassemia departments, the intermediate care unit, etc.

### 4.2. Bacterial Identification and Antimicrobial Susceptibility Testing

Pathogen identification was performed using the VITEK 2 system (bioMerieux, Marcy-l’Étoile, France). Antimicrobial susceptibility testing (AST) of antibiotic agents, including ciprofloxacin, levofloxacin, piperacillin/tazobactam, cefepime, ceftazidime, ceftazidime/avibactam, aztreonam, imipenem, meropenem, gentamicin, tobramycin, amikacin, and colistin, was performed either using VITEK 2 or the disk diffusion method, as described by EUCAST 2020 guidelines [34]. Colistin resistance was verified by the reference method of broth microdilution. Resistance rates were determined based on EUCAST breakpoint criteria. Specifically, bacterial isolates were classified as resistant or susceptible according to their minimum inhibitory concentration (MIC) values.

### 4.3. Detection of Carbapenemases

For the detection and differentiation of the five most prevalent carbapenemases (KPC, VIM, IMP, NDM, and OXA-48), a lateral flow immunoassay (LFIA) (NG-Test CARBA5, NG Biotech, Guipry-Messac, France) and FilmArray system (bioMerieux, Marcy l’Etoile, France) were used, with the latter applied only to blood cultures, according to the manufacturer’s recommendations.

### 4.4. Statistics

Statistical analysis was performed using IBM SPSS Statistics (Version 25) [35]. Categorical variables were compared using the chi-square test, with statistical significance set at a *p*-value < 0.05. To evaluate changes in antimicrobial resistance over the study period, pairwise comparisons were conducted between consecutive years (2020 vs. 2021, 2021 vs. 2022, and 2022 vs. 2023) and between 2020 vs. 2023. Resistance rates were expressed as percentages of resistance isolates out of the total isolates tested for each antibiotic annually. Spearman’s correlation analysis was used to evaluate the association between the years and the total number of CPPA infections per year. Statistical significance was set at a *p*-value of 0.05.

## 5. Conclusions

In this study, the emergence of NDM-positive CPPA isolates was observed in 2022, surpassing the rate of VIM-positive isolates by 2023. The resistance rates to almost all tested antibiotics remained high, with the exception of colistin and aztreonam. These findings highlight the urgent need for continuous epidemiological surveillance, robust antimicrobial stewardship measures, and the introduction of new antibiotics and antibiotic combinations.

## Figures and Tables

**Table 1:** Distribution of Isolates by sample type across study years.

Type of Samples (%)	2020	2021	2022	2023	2020–2023
Blood culture	9 (9%)	5 (5%)	9 (7%)	22 (7%)	45 (7%)
Bronchial secretions	35 (34%)	39 (37%)	17 (14%)	37 (12%)	128 (20%)
CVC *	4 (4%)	4 (4%)	7 (6%)	15 (5%)	30 (5%)
Urine	23 (23%)	36 (34%)	54 (44%)	131 (44%)	244 (39%)
Wound swabs	15 (15%)	12 (11%)	11 (9%)	46 (15%)	84 (13%)
Multiple sample areas	16 (16%)	8 (8%)	22 (18%)	42 (14%)	88 (14%)
Other **	0 (0%)	1 (1%)	3 (2%)	5 (2%)	9 (1%)
Total	*n* = 102	*n* = 105	*n* = 123	*n* = 298	628

* CVC (Central Venous Catheter), ** “other” category includes drainage fluids, aspirates, and eyes.

**Table 2:** Distribution of carbapenemase-producing *P. aeruginosa* isolates by hospital department.

	2020	2021	2022	2023	2020–2023
Pathology	10 (10%)	18 (17%)	38 (31%)	92 (31%)	158 (25%)
ICU *	51 (50%)	44 (42%)	11 (9%)	35 (12%)	141 (22%)
Surgery	28 (27%)	19 (18%)	20 (16%)	48 (16%)	115 (18%)
AED **	2 (2%)	5 (5%)	17 (14%)	38 (13%)	62 (10%)
Orthopedics	0 (0%)	1 (1%)	5 (4%)	6 (2%)	12 (2%)
Hematology	1 (1%)	2 (2%)	3 (2%)	4 (1%)	10 (2%)
AED-COVID	0 (0%)	0 (0%)	6 (5%)	13 (4%)	19 (3%)
Nephrology/Urology	3 (3%)	10 (10%)	3 (2%)	10 (3%)	26 (4%)
Others ***	7 (7%)	6 (6%)	20 (16%)	52 (17%)	85 (14%)
Total	102	105	123	298	628

* ICU (Intensive Care Unit), ** AED (Accident and Emergency Department), *** “other” category (including gastroenterology, neurology, rheumatology, ophthalmology, thalassemia department, intermediate care unit, etc.).

**Table 3:** Carbapenemase-producing *P. aeruginosa* for each year studied.

	2020	2021	2022	2023
VIM	102 (100%)	105 (100%)	70 (57%)	136 (45.6%)
NDM	0 (0%)	0 (0%)	53 (43%)	162 (54.4%)
Total	*n* = 102	*n* = 105	*n* = 123	*n* = 298

**Table 4:** Antimicrobial resistance trends in *P. aeruginosa*. (2020–2023).

Antibiotic	Resistance	2020 vs. 2021	2021 vs. 2022	2022 vs. 2023	2020 vs. 2023
Piperacillin/Tazobactam	2020—78/97 (80.4%)	*p*-value = < 0.00001	NS	NS	*p*-value = < 0.00001
	2021—105/105 (100%)				
	2022—123/123 (100%)				
	2023—298/298 (100%)				
Ceftazidime	2020—79/102 (77.5%)	*p*-value = < 0.00001	NS	NS	*p*-value = < 0.00001
	2021—105/105 (100%)				
	2022—123/123 (100%)				
	2023—297/297 (100%)				
Cefepime	2020—87/102 (85.3%)	*p*-value = 0.000797	NS	NS	*p*-value = < 0.00001
	2021—103/105 (98.1%)				
	2022—122/123 (99.2%)				
	2023—295/298 (99.0%)				
Aztreonam	2020—17/101 (16.8%)	NS	*p*-value = 0.015737	NS	*p*-value = < 0.00001
	2021—26/105 (24.8%)				
	2022—49/123 (39.8%)				
	2023—124/296 (41.9%)				
Imipenem	2020—102/102 (100%)	NS	NS	NS	NS
	2021—105/105 (100%)				
	2022—123/123 (100%)				
	2023—298/298 (100%)				
Meropenem	2020—101/102 (99.0%)	NS	NS	NS	NS
	2021—102/105 (97.1%)				
	2022—122/123 (99.2%)				
	2023—297/298 (99.7%)				
Gentamicin	2020—80/100 (80.0%)	*p*-value = 0.000377	NS	NS	*p*-value = < 0.00001
	2021—99/103 (96.1%)				
	2022—107/114 (93.9%)				
	2023—261/268 (97.4%)				
Tobramycin	2020—98/102 (96.1%)	NS	NS	NS	NS
	2021—103/105 (98.1%)				
	2022—120/121 (99.2%)				
	2023—203/208 (97.6%)				
Amikacin	2020—38/45 (84.4%)	NS	*p*-value = 0.019517	*p*-value = 0.018257	NS
	2021—87/95 (91.6%)				
	2022—118/120 (98.3%)				
	2023—275/298 (92.3%)				
Ciprofloxacin	2020—90/98 (91.8%)	*p*-value = 0.012626	NS	NS	NS
	2021—104/105 (99.0%)				
	2022—119/122 (97.5%)				
	2023—267/282 (94.7%)				
Levofloxacin	2020—87/94 (92.6%)	NS	NS	NS	NS
	2021—104/105 (99.0%)				
	2022—120/123 (97.6%)				
	2023—281/298 (94.3%)				
Colistin	2020—3/102 (2.9%)	NS	NS	NS	NS
	2021—2/105 (1.9%)				
	2022—2/123 (1.6%)				
	2023—8/298 (2.7%)				
Ceftazidime/Avibactam	2020-				
	2021—2/2 (100%)				
	2022—60/60 (100%)				
	2023—269/269 (100%)				

NS: non significant.

**Table 5:** Antimicrobial resistance to the main antibiotics for *P. aeruginosa.* stratified by the type of carbapenemase during the four-year period studied.

	2020	2021	2022		2023		2020–2023	
Antimicrobial	VIM	VIM	VIM	NDM	VIM	NDM	VIM	NDM
Piperacillin/Tazobactam	78/97 (80%)	105/105 (100%)	70/70 (100%)	53/53 (100%)	136/136 (100%)	162/162 (100%)	389/408 (95.3%)	215/215 (100%)
Ceftazidime	79/102 (77%)	105/105 (100%)	70/70 (100%)	53/53 (100%)	136/136 (100%)	161/161 (100%)	390/413 (94.4%)	214/214 (100%)
Cefepime	87/102 (85%)	103/105 (98%)	69/70 (99%)	53/53 (100%)	133/136 (98%)	162/162 (100%)	392/413 (94.9%)	215/215 (100%)
Aztreonam	17/101 (17%)	26/105 (25%)	21/70 (30%)	28/53 (53%)	34/135 (25%)	90/161 (56%)	98/411 (23.8%)	118/214 (55.1%)
Gentamicin	80/100 (80%)	99/103 (96%)	58/64 (91%)	49/49 (100%)	114/121 (94%)	147/147 (100%)	351/388 (90.5%)	196/196 (100%)
Tobramycin	98/102 (96%)	103/105 (98%)	69/70 (99%)	51/51 (100%)	92/95 (97%)	111/113 (98%)	362/372 (97.3%)	162/164 (98.8%)
Amikacin	38/45 (84%)	87/95 (92%)	66/68 (97%)	52/52 (100%)	116/136 (85%)	159/162 (98%)	307/344 (89.2%)	211/214 (98.6%)
Ciprofloxacin	90/98 (92%)	104/105 (99%)	67/70 (96%)	52/52 (100%)	110/125 (88%)	157/157 (100%)	371/398 (93.2%)	209/209 (100%)
Levofloxacin	87/94 (93%)	104/105 (99%)	67/70 (96%)	53/53 (100%)	120/136 (88%)	161/162 (99%)	378/405 (93.3%)	214/215 (99.5%)
Colistin	3/102 (3%)	2/105 (2%)	2/70 (3%)	0/53 (0%)	1/136 (1%)	7/162 (4%)	8/413 (1.9%)	7/215 (3.3%)
Ceftazidime/Avibactam	-	2/2 (100%)	28/28 (100%)	32/32 (100%)	122/122 (100%)	14/147 (100%)	152/152 (100%)	179/179 (100%)

## Data Availability

The original contributions presented in this study are included in the article. Further inquiries can be directed to the corresponding author(s).
